# Psoriatic arthritis digital phenotyping and inflammation drivers (PDPID) study: protocol for an international multicentre prospective cohort

**DOI:** 10.1136/bmjopen-2025-115903

**Published:** 2026-09-28

**Authors:** Batoul Hojeij, Ilja Tchetverikov, Eleni Vasileiou, George Apostolidis, Theodoros Dimitroulas, Maria Mytilinaiou, Cátia Gonçalves, Ana M Rodrigues, Laura Coates, Dimitrios Konstantinidis, Kosmas Dimitropoulos, Nikos Melanitis, Jasper Foolen, Wendy Wagenaar, Vasileios Charisis, Leontios J Hadjileontiadis, Jolanda J Luime

**Affiliations:** 1 Department of Rheumatology, Erasmus MC, Rotterdam, The Netherlands; 2 Department of Rheumatology, Albert Schweitzer Hospital, Dordrecht, The Netherlands; 3 Department of Electrical & Computer Engineering, Aristotle University of Thessaloniki, Thessaloniki, Greece; 4 Department of Internal Medicine, Hippokrateion Hospital, Thessaloniki, Greece; 5 Comprehensive Health Research Center (CHRC), NOVA Medical School, Lisbon, Portugal; 6 Reuma.pt, Sociedade Portuguesa de Reumatologia, Lisbon, Portugal; 7 Unidade de Reumatologia, Hospital dos Lusíadas, Lisbon, Portugal; 8 Rheumatology and Musculoskeletal Sciences (NDORMS), University of Oxford, Oxford, UK; 9 Information Technologies Institute, Centre for Research and Technology-Hellas, Thessaloniki, Greece; 10 Innovation & Incubation Center KU Leuven, AINIGMA Technologies, Leuven, Belgium; 11 Department of Biomedical Engineering, Eindhoven University of Technology, Eindhoven, The Netherlands; 12 Tranzo, Tilburg University, Tilburg, The Netherlands

**Keywords:** Inflammation, RHEUMATOLOGY, Artificial Intelligence

## Abstract

**Abstract:**

**Introduction:**

Changes in psoriatic arthritis (PsA) disease activity, particularly flares, are unpredictable and can impact patients’ quality of life. Digital biomarkers derived from data collected via smart devices provide an opportunity for unobtrusive continuous monitoring of symptoms reflecting disease activity. Besides, our understanding of the mechanisms and triggers behind flares is limited. The primary objectives of the Psoriatic Arthritis Digital Phenotyping and Inflammation Drivers (PDPID) study are: (1) to develop digital biomarker capable of detecting PsA flare using data collected from patient’s smartphone and smartwatch, and (2) to develop machine learning models for PsA flare prediction using clinical, biological, environmental and digital data.

**Methods and analysis:**

The PDPID study is a 12-month multi-centre prospective cohort study conducted across four countries. Study visits are scheduled at baseline (T0), and 3, 6, 9 and 12 months follow-up visits, with additional patient-initiated visits due to flare. At inclusion, patients have a study app installed on their smartphone and receive a smartwatch. Flare information is collected using physician- and patient-reported flare questionnaires administered at each visit, and via the flare button in the app that patients can activate. The study collects clinical data (physical measurements, questionnaires), biological markers (salivary DNA, gut microbiome, hair cortisol, C-reactive protein) and environmental exposure data (weather and air pollution). The app includes in-app questionnaires, active photo and video tests and passively captures digital data from the smartphone and smartwatch sensors.

**Ethics and dissemination:**

Ethical approval has been granted from each of the participating countries (Erasmus MC, the Netherlands: MEC-2023-0470; HRA and Health and Care Research Wales, UK: 332916 NOVA Medical School|Faculdade de Ciências Médicas (NMS|FCM) Ethical Committee (CEFCM), Portugal (124/2023/CEFCM); MREC Hipokrateion Hospital Thessaloniki, Greece: 5549/31.01.24). Findings of this study will be disseminated through reports to the funding body, the project website, newsletters, social media, national and international conferences and symposiums, and scientific publications.

**Trial registration number:**

NCT06347237.

**STRENGTHS AND LIMITATIONS OF THIS STUDY**

- This study will develop digital biomarker for continuous unobtrusive monitoring of psoriatic arthritis (PsA) disease activity.
- Identification of triggers of PsA flare will be conducted using multimodal data including clinical, biological, environmental and digital measures.
- Diverse PsA patient population from multiple centres across four countries and in real-world clinical settings.
- Prospective collection of physician- and patient-reported flare during the study and patient-initiated visits, and patient-reported flare registered via the app.
- Limitations associated with the study include participant dropout, loss to follow-up, missing data, recall bias in questionnaires and external validity of the findings.

## Introduction

 Psoriatic arthritis (PsA) is a chronic, immune-mediated inflammatory arthritis associated with psoriasis (PsO). The prevalence of PsA in the adult general population is around 0.1% and it affects 20% of patients with PsO.^1 2^ The disease manifestations can be heterogeneous between subjects, and the resulting musculoskeletal impairment can interfere with physical function as well as the quality of life of patients.^3 4^ Depending on disease activity, patients can experience burden at the physical, psychological, social and financial levels.^5–8^

The pathogenesis of PsA involves a complex interplay between genetic predisposition and environmental triggers, and results in dysregulated immune-mediated inflammatory processes.^9^ Depending on how PsA disease activity is manifested, it primarily affects joints causing pain, fatigue and stiffness, but may also affect sleep, mood and stress levels.^6 10–14^ In turn, factors including pain, fatigue, sleep, mood and stress can also influence the immune-inflammatory pathways, acting as both triggers and consequences of changes in disease activity.^15–19^ Genetic factors may also contribute to PsA disease activity; however, this area is under investigated.^20–22^ For example, genetic polymorphisms in genes such as TRAF3IP2, IL1β and TNF-α have been associated with increased disease activity.^20–22^ Of interest, studying genetic factors helps us better understand interpatient differences in pain and treatment response, and susceptibility to different disease phenotypes.^23–25^ The gut microbiome has also gained attention lately in PsA due to its role in regulating the immune-inflammatory processes.^26 27^ Immuno-inflammatory pathways may also be influenced by environmental exposures such as weather conditions and air pollution, although data in PsA is very scarce.^28–31^ Currently, factors that trigger changes in PsA disease activity are not well understood.^32^ Knowledge of these triggers can lead to better PsA management.

PsA disease activity is assessed during routine clinical visits using physical examinations and patient-reported outcomes (PROs).^33^ Although this is standard practice, it provides limited information into disease activity changes between visits, especially when those visits are infrequent. Importantly, exacerbations in PsA disease activity, known as flares, are usually unpredictable, which emphasises the need for continuous (daily) monitoring of patients.^32 34^ The use of questionnaires to collect PROs during visits or to monitor disease activity between visits is subject to recall bias and survey fatigue, which compromise the reliability and completeness of responses.^35 36^ Moreover, frequent use of questionnaires can increase the burden on the patient.

Instead, smart devices such as smartphones or smartwatches contain sensors (eg, motion sensors and touch screens) that collect passive real-time data. This data can be translated into digital biomarkers, allowing for continuous and unobtrusive monitoring of the user’s health.^37 38^ These monitoring capabilities combined with the widespread use of these devices (particularly smartphones) make them a cost-effective and less burdensome tool for remote health monitoring in clinical settings.^37^ With respect to arthritis, changes in disease activity can influence movement patterns and other disease manifestations such as pain, fatigue and sleep disturbances, all of which can be captured using digital biomarkers.^10 12 13 34 39–42^ The ActConnect study also showed promising results for detecting rheumatic disease flares using digital biomarkers.^34 43^ However, to the best of our knowledge, no previous studies examined digital biomarkers for remote monitoring of PsA disease activity.

### Objectives

The primary objectives of the Psoriatic Arthritis Digital Phenotyping and Inflammation Drivers (PDPID) study are to develop and internally validate (1) a digital biomarker capable of detecting flare in patients with PsA using data collected from patient’s smartphone and smartwatch, benchmarked against clinically defined flares as assessed by the rheumatologist, and (2) machine learning models for flare prediction based on clinical, gut microbiome, genetic and environmental data collected prospectively. The primary and secondary study objectives are detailed in table 1.

**Table 1:** Psoriatic arthritis digital phenotyping and inflammation drivers (PDPID) study objectives

Objective type	Objective description
Primary objective 1	Develop and internally validate a machine learning model for detecting flares in patients with PsA using accelerometer data, keystroke dynamics and screen time metrics (ie, digital biomarker) to assess changes in physical activity patterns against clinically defined flare by the rheumatologist
Primary objective 2	Develop and internally validate machine learning models for flare prediction in patients with PsA that capitalise on sleep, fatigue, pain, stress, mechanical stress, gut microbiome composition, genetic predisposition and environmental exposure
Secondary objective 1	Assess the construct validity of the machine learning model for detecting flare in patients with PsA patients against clinical composite scores of disease activity and impact of disease
Secondary objective 2	Develop and internally validate a machine learning model for changes in joint and skin appearance that relates to flare in patients with PsA patients using video analysis of hand, posture and gesture and photos of the hands and feet, against clinically defined flare by the rheumatologist
Secondary objective 3	Determine the intraperson reliability of the artificial intelligence (AI)-driven digital biomarker system
Secondary objective 4	Determine clinically relevant changes in the AI-driven digital biomarker system
Secondary objective 5	Determine minimal detectable difference in the AI-driven digital biomarker system
Secondary objective 6	Assess the intraperson variation of stress, mechanical stress and changes in gut microbiome on the occurrence of flare
Secondary objective 7	Assess genetic contribution to disease activity and pain
Secondary objective 8	Evaluate costs and effects of the digital biomarker
Secondary objective 9	Evaluate the compliance and satisfaction of the users with the digital measurement of disease activity and flare

PsA, psoriatic arthritis.

## Methods and analysis

### Study design and population

Initiated in September 2024, the PDPID study is an international, multicentre, prospective observational cohort study recruiting patients diagnosed with PsA. The study is conducted across 30 sites in four European countries (the Netherlands, UK, Portugal and Greece) participating in the PsA inflammation explained through multi-source data analysis guiding a novel personalised digital care ecosystem (iPROLEPSIS) project^44^ and representing North-Western and Southern Europe.

Patients with PsA are invited by their rheumatologist to participate in the PDPID study during their routine clinical consultation at the Rheumatology outpatient clinic of the participating centres. If the patient is willing to participate, they are assessed for eligibility based on the inclusion and exclusion criteria. Those who meet the inclusion criteria are provided with a copy of the participant information sheet (PIS) which includes the informed consent. Patients may also be contacted by phone and provided with the PIS via email or post. Within 7 days, the research team contacts the potential participant to ask whether they wish to join the study. If the patient agrees to participate, the first study visit is planned (T0). During this visit, the patient receives detailed information about the study and signs the consent form. At some sites, an invitation letter from the treating rheumatologist is sent to the patient’s home address to inform them about the study and explain how they can contact the research team if they wish to participate.

Patients are included if they meet the following criteria: (1) 18 years or older and competent (ie, capable of providing informed consent), (2) diagnosed with PsA, (3) use a smartphone, (4) agree to use a smartwatch and (5) have a good command of the local language. Patients are excluded if they are: (1) less than 18 years and (2) incapacitated (ie, incapable of providing informed consent).

Patients may withdraw from the study at any time without providing a reason and this will not affect their standard of care.

### Observation period

Patients will be followed for a total duration of 12 months. A baseline visit will be conducted at inclusion, followed by four follow-up visits every 3 months (figure 1).

**Figure 1:**
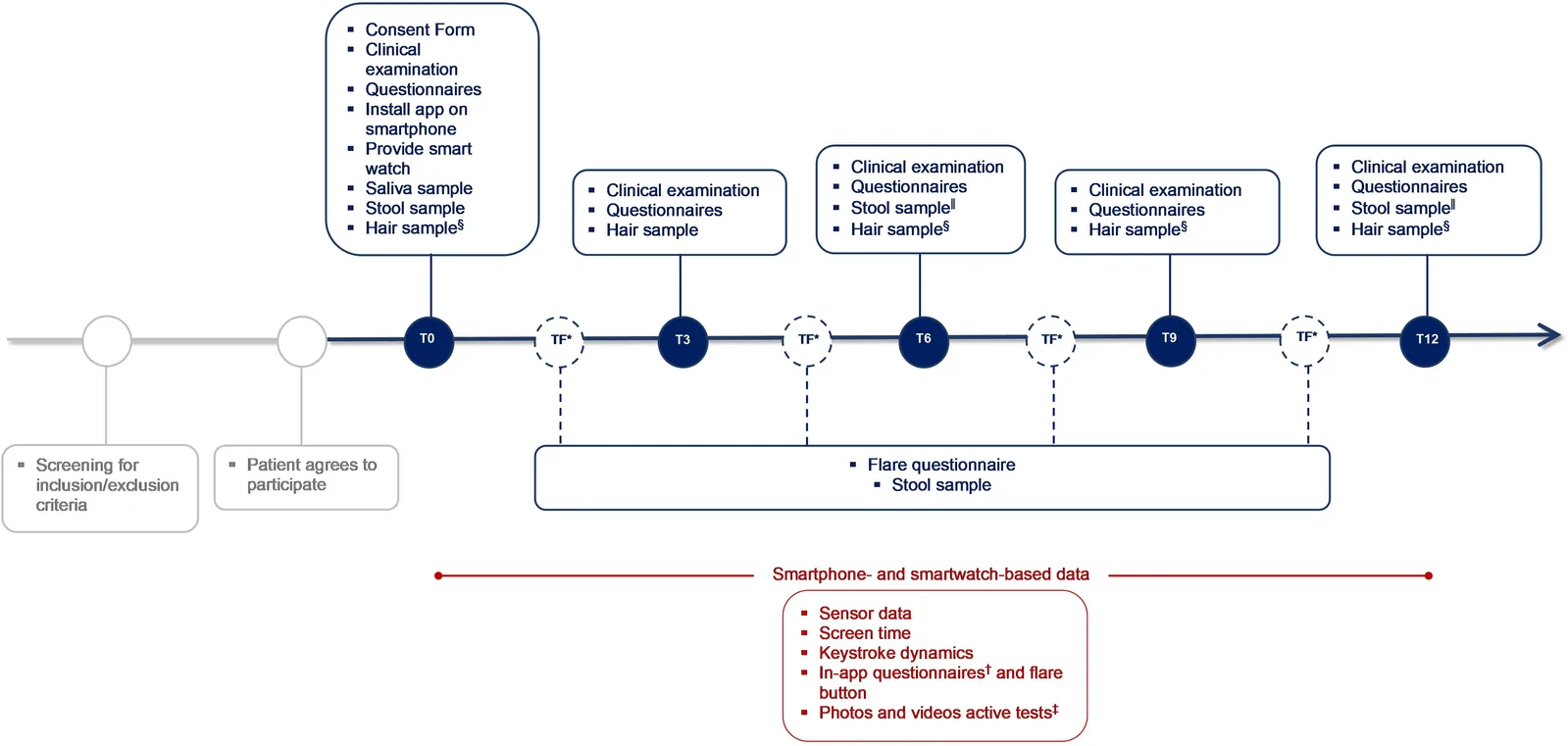
Psoriatic Arthritis Digital Phenotyping and Inflammation Drivers (PDPID) study procedures. ^*^TF (T flare) is an additional visit if the participant attends the clinic and seeks help from the rheumatologist due to a flare. ^§^Hair sample collection at T0, T6, T9 is optional if the participant chooses to. ^‖^Additional stool sample collection at T6 and T12 from participants in Netherlands only. †Daily in-app questionnaire is completed for the first 14 days after inclusion and when the flare button is ON. ^‡^Photos and videos active tests are performed every 6 weeks.

### Sample size calculation

#### Detection of flare

We estimate that between 20% and 50% of PsA patients will experience a clinically detectable flare within 1 year of disease monitoring.^43^ At the time of these calculations, test performance of the AI-driven digital biomarker was unknown, thus we aimed for a sensitivity of 90% and a specificity of 80% of the digital biomarker using a conservative estimate that 25% of patients with PsA would experience a flare within 1 year. Based on these assumptions, we would require 554 patients, allowing for 10% width around the estimate.^45^

#### Prediction of flare

We used the generally accepted rule of thumb of 10 patients per event with a 25% prevalence of flare, which yielded 554 patients. Among the 554 patients, 139 patients (25%) are expected to experience flare, allowing for the inclusion of 12 variables plus the intercept in the prediction model.^46 47^ These variables include age, sex, initial disease activity state, sleep, stress, keystroke dynamics-derived features, accelerometer-based activity features and screen time.

### Study procedures

The study procedures are depicted in figure 1. Briefly, after the patient agrees to participate and signs the informed consent form, the baseline visit will include: clinical examination, completion of questionnaires, collection of biological samples (stool, saliva and optionally hair), installation of the study app on the patient’s phone and provision of a smartwatch to be worn daily, which will be connected to the app during the visit. The app collects sensor data from the smartphone and smartwatch, keystroke dynamics from the smartphone’s virtual keyboard, smartphone screen time, and includes a flare button, in-app questionnaires and photo and videos active tests. Following the baseline assessment, patients will be followed up every 3 months for a total of one year. During the follow-up visits, patients will undergo a clinical examination, complete questionnaires, have their app and smartwatch checked and are asked to provide a hair sample. Additionally, a patient-initiated visit may occur if the participant seeks care at the outpatient clinic due to a disease flare. During this flare visit, the treating physician will perform an assessment according to standard care procedures, and a flare questionnaire will be completed and the participant is asked to provide a further stool sample.

#### Questionnaires

A summary of the collected questionnaires and their collection timeline is presented in table 2. Questionnaires are completed during the study visits and remotely via online surveys or telephone interviews, and all responses are recorded in the electronic case report forms. The questionnaires are administered at baseline and at each follow-up visit every 3 months covering general and clinical overview, medication (anti-rheumatic and non-anti-rheumatic) and dietary supplements, flare, disease symptoms, quality of life, lifestyle and psychological factors, socio-economic impact, biological samplings, digital literacy and app user experience. If a participant visits the outpatient clinic due to a flare, both patient- and physician-reported flare data are collected using the flare questionnaires.

**Table 2:** Clinical collected measures of the PDPID study

Collected measure	Parameter	Assessment period					
		T0	T3	T6	T9	T12	TFlare
General and clinical overview							
Socio-demographic factors	Other	✓					
Occupational factors	Other	✓					
Disease duration	Other	✓					
Global rating of change	Other	✓	✓	✓	✓	✓	
Patient comorbidities	Other	✓				✓	
C-section birth	Other	✓					
Medication and supplement use							
Anti-rheumatic drugs	Other	✓	✓	✓	✓	✓	
Non anti-rheumatic drugs	Other	✓	✓	✓	✓	✓	
Dietary supplements	Other	✓	✓	✓	✓	✓	
Physical measurements							
Swollen and tender joint count	Other	✓	✓	✓	✓	✓	
Enthesitis	Other	✓	✓	✓	✓	✓	
Dactylitis	Other	✓	✓	✓	✓	✓	
Psoriasis	Other	✓	✓	✓	✓	✓	
CASPAR	Other	✓					
Disease phenotype	Other	✓					
PGA	Other	✓	✓	✓	✓	✓	
Anthropometrics	Other	✓					
Flare							
Remission	Primary	✓	✓	✓	✓	✓	✓
Low disease activity	Primary	✓	✓	✓	✓	✓	✓
Patient acceptable symptom state	Primary	✓	✓	✓	✓	✓	✓
Flare	Secondary	✓	✓	✓	✓	✓	✓
Disease activity composite scores							
PASDAS	Secondary	✓	✓	✓	✓	✓	
DAPSA	Secondary	✓	✓	✓	✓	✓	
MDA	Secondary	✓	✓	✓	✓	✓	
Patient-reported symptoms							
PSAID	Secondary	✓	✓	✓	✓	✓	
Morning stiffness	Secondary	✓	✓	✓	✓	✓	
Pain VAS	Other	✓	✓	✓	✓	✓	
PtGA	Other	✓	✓	✓	✓	✓	
Quality of life							
HAQ	Other	✓	✓	✓	✓	✓	
SF-36	Other	✓	✓	✓	✓	✓	
EQ-5D	Other	✓	✓	✓	✓	✓	
Psychological factors							
PHQ9	Other	✓	✓	✓	✓	✓	
Life satisfaction	Other	✓	✓	✓	✓	✓	
Life events	Other	✓				✓	
Lifestyle factors							
Smoking	Other	✓					
Alcohol	Other	✓					
Physical activity	Other	✓					
Perceived stress	Other	✓					
Diet	Other	✓					
Productivity and healthcare utilisation							
WPAI	Other	✓	✓	✓	✓	✓	
Healthcare usage	Other	✓	✓	✓	✓	✓	
Digital skills and user experience							
Handedness and typing fingers	Other	✓					
Usability	Other			✓		✓	
Digital literacy	Other	✓					
Biological markers							
DNA	Other	✓					
Gut microbiome	Other	✓		✓*		✓*	✓
Cortisol	Other	✓†	✓	✓†	✓†	✓†	
C-reactive protein	Other	✓	✓	✓	✓	✓	

* Collected from patients included in the Netherlands.

† Optional if the patient chooses to.

CASPAR, classification criteria for psoriatic arthritis; DAPSA, disease activity in psoriatic arthritis; EQ-5D, EuroQol Five-Dimension Questionnaire; HAQ, health assessment questionnaire; MDA, minimal disease activity; PASDAS, psoriatic arthritis disease activity score; PGA, physician global assessment; PHQ-9, patient health questionnaire-9; PSAID, psoriatic arthritis impact of disease; PtGA, patient global assessment; SF-36, short form (36) health survey; VAS, visual analogue scale; WPAI, work productivity and activity impairment.

#### Clinical examinations

Classification criteria for psoriatic arthritis (CASPAR) criteria, PsA phenotype and anthropometric measures are assessed at baseline (table 2). Physical measurements for PsA (swollen and tender joint count, enthesitis, dactylitis, PsO) are performed at baseline and each follow-up visit every 3 months (table 2).

#### Biological markers

A list of the biological markers and their collection timeline is provided in table 2. Blood C-reactive protein levels are obtained at each study visit from the patient medical records at the local laboratories of the participating centres as part of the routine clinical. For DNA analysis, patients are asked to provide a saliva sample in a collection tube during the baseline visit. Patients are provided with a stool collection kit to collect stool samples at home at baseline and when the participant experiences flare, to analyse their gut microbiome. Patients attending centres in the Netherlands are asked to provide an additional stool sample at months 6 and 12. Hair samples are collected at the 3-month visit to measure cortisol levels as an indicator of stress over the past months. Providing hair samples at baseline and at months 6, 9 and 12 is optional if the participant chooses to.

#### Smartphone application and smartwatch

The miPROLEPSIS app is installed on the patient’s smartphone during the baseline visit. It functions as a data collection tool, gathering data from the smartphone and smartwatch. The app can be downloaded from the iOS App Store and Google Play Store, and is available in four languages (Dutch, English, Greek and Portuguese). During the baseline visit, patients receive a smartwatch (Garmin Vivoactive 5), which is paired with the miPROLEPSIS app, and used throughout the study period to support continuous participant monitoring.

Table 3 presents a summary of the digital data, their source and corresponding timeline. Briefly, the app collects data on flare via a toggle button to self-register a disease flare, PROs via in-app questionnaires and passive digital measures from smartphone and/or smartwatch (inertial measurement unit (IMU) sensors, keystroke dynamics, screen time, physical activity and wellness metrics) (figure 2). Daily in-app questionnaires are automatically sent during the first 14 days after inclusion. The questionnaires cover sleep quality, morning stiffness, pain, fatigue, functional capacity, skin symptoms and mood, and include the Psoriasis Epidemiology Screening Tool (PEST) joint manikin which allows patients to indicate joints causing discomfort. After this period, if the participant registers a flare in the app by activating the flare button, the daily questionnaires resume and continue for as long as the flare button is activated. When patients do not register a flare in the app, a control questionnaire is sent every 2 weeks to confirm that no flare was experienced (table 3). Using the phone camera, the app also collects images of both hands and feet as well as videos of movement tests capturing hand gestures and upper- and lower-body postures, at baseline, every 6 weeks and when the participant registers a flare in the app. For the video tests, an additional companion application (miPROLEPSIS Joint Landmarker) is downloaded and automatically launched by the miPROLEPSIS app when the user starts the movement assessments. Only the derived time series of hand and body landmarks are stored to compute skeletal data (raw video footage is not saved).

**Table 3:** Digital collected measures of the psoriatic arthritis digital phenotyping and inflammation drivers (PDPID) study

Collected measure	Baseline (daily for first 2 weeks)	PtFlare (daily while flare button is ON)	Follow-up		Continuous (unobtrusive)
			2 weeks interval	6 weeks interval	
App measures					
Flare button for PtFlare		✓			
NRS for pain, fatigue, morning stiffness, sleep, functional capacity, skin psoriasis, happiness	✓	✓			
PEST	✓	✓			
Global rating of change			✓		
Flare recall			✓		
Photos for hand and feet				✓	
Videos for hand, gesture and posture				✓	
Smartphone measures					
Accelerometer					✓
Gyroscope					✓
Keyboard (keystroke dynamics)					✓
Screen time					✓
Smartwatch measures					
Accelerometer					✓
Physical activity (intensity and category)					✓
Motion intensity					✓
Steps					✓
Distance					✓
Body battery					✓
Stress					✓
Heart rate					✓
Beat-to-beat intervals					✓
Sleep metrics and staging					✓

Digital parameters are part of the other study parameters.

NRS, numerical rating scale; PEST, Psoriasis Epidemiology Screening Tool; PtFlare, patient-reported flare.

**Figure 2:**
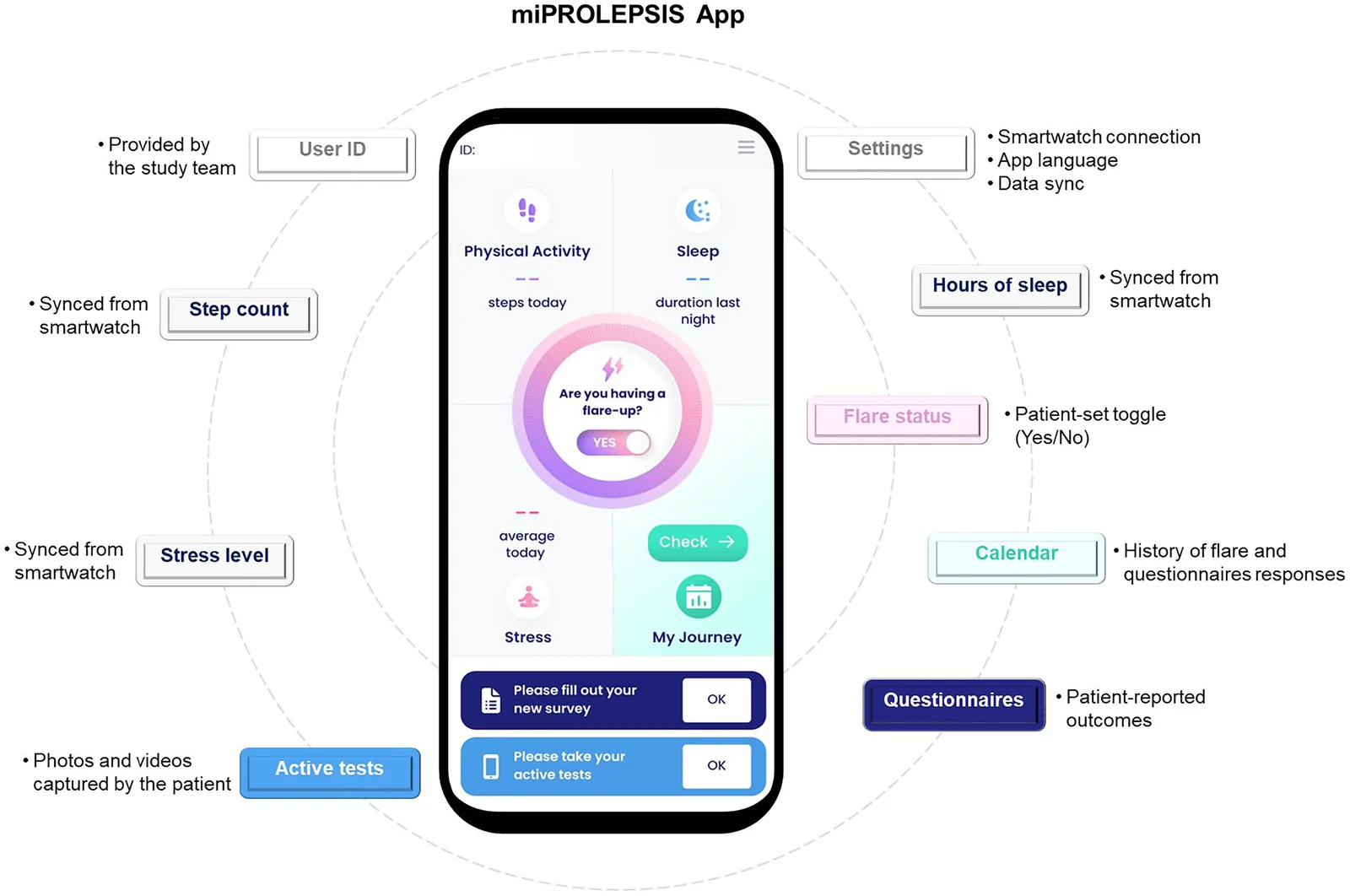
miPROLEPSIS smartphone application.

All data collected by the miPROLEPSIS app is available to study participants.

#### Environmental data

Weather and air pollution exposures are assessed at the residential and occupational addresses using the participant postal codes collected at the baseline visit. These environmental exposures are obtained from open-source databases, based on measurements from monitoring stations located in the vicinity of the corresponding postal codes for each patient.

### Patient and public involvement

We work together with PsA patient partners to develop methods for improving early flare detection (eg, using digital biomarkers), and to gain insights into patients’ perspectives and improve knowledge of flares.^48^ During the study design, patients with PsA tested the app features and provided feedback; and throughout the study, participants complete a usability questionnaire to assess the app usability and user experience. In addition, we have patient partners with whom we discuss the study objectives, procedures and progress to ensure that the research is patient-centred.

### Data analysis plan

Descriptive statistics (eg, mean and standard deviations, median and interquartile ranges, frequencies and percentages) will be used to present the characteristics of the study population. Group differences will be analysed using parametric or non-parametric tests for continuous variables, and χ^2^ test for categorical variables.

To study the associations between potential risk factors and triggers of PsA inflammation, crude and multivariable statistical analysis methods will be used. The choice of the model will depend on the type of data being analysed. For example, linear and logistic regression models will be used for continuous and categorical outcomes, respectively; mixed-effects models will be used for repeated measures outcomes; Cox proportional hazard models will be used for time-to-event analyses.

Typing sessions captured by the smartphone keyboard will include transient features (key hold time, flight time, typing speed and normalised pressure) and metadata-derived features (session duration, total characters typed and deletion rate). Continuous accelerometer data collected during each session will provide complementary information on device motion and orientation. All metrics will be processed into structured feature vectors representing each typing session and analysed using machine learning models to identify patterns indicative of impaired finger mobility, reduced dexterity or altered fine motor control. The raw inertial data captured by the smartphone and smartwatch (tri-axial accelerometer and gyroscope signals) will be processed to extract features reflecting physical activity patterns, gait, and postural states. Smartphone-derived features will include walking activity counts, cadence, and gait characteristics, while smartwatch-derived features will represent daily behavioural patterns such as sleep, morning activity, activity intensity and volume, fragmentation and walking metrics (eg, step count and cadence). These features will be aggregated into structured vectors and analysed using machine learning models to identify patterns associated with changes in physical function, pain, stiffness, sleep quality, disease impact and PsA disease activity. The resulting digital biomarkers will be quantified into continuous scores and evaluated against clinically defined PsA disease activity, flare status and relevant PROs, ultimately contributing to the detection and monitoring of PsA disease activity and flares.

Test-retest reliability of the digital biomarkers will be determined by assessing the degree of similarity between repeated features produced over a pre-specified period using intraclass correlation coefficient values. Construct validity of the digital biomarker will be evaluated against self-registered flare in the app, and physician- and patient-reported flare from the flare questionnaire. In addition, the PSAID with area under the curve (AUC) will be used with different cut-offs for precision and recall. Change in the digital biomarker will be correlated with changes in disease symptoms (eg, pain, fatigue, stiffness, sleep), self-registered flare in the app and clinical markers of disease activity. Anchor-based and distribution-based methods will be adopted to triangulate the ranges of minimal important changes (MIC). The distribution-based MIC estimate is determined using Cohen’s effect size benchmark. Minimal detectable difference will be determined based on the bandwidth.

Images of hands and feet will be processed with image processing and computer vision techniques to generate feature vectors relative to the condition of nails and joints. Metrics (eg, joint thickness estimation, nail classification) will be calculated for each finger and toe, and adjusted for skin tone bias using the Fitzpatrick Skin Tone classification. For videos, each recording will be processed frame-by-frame to detect a set of body and hand joint landmarks, and their coordinates will be tracked across subsequent video frames. The output will be a set of time series with landmark coordinates. From these time series, the range-of-motion (RoM), ie, the angle in degrees travelled by a joint during an exercise, for each joint of interest will be computed. These RoM measurements will then be correlated with corresponding RoM measurements from a healthy population, and scores will be computed to measure the deviation between a patients’ test execution and a reference model of performance. The exact set of scores will depend on the specific test performed, while aggregated scores from all different tests will also be computed. Additional data that will be collected are the total number of successful test repetitions, as well as the number of missing test performances, indicating potential difficulties in performing these tests. To process the video recordings and acquire the corresponding time series of landmarks, existing open-source software tools (eg, MediaPipe) will be employed, while smoothing and interpolation postprocessing techniques will be used to compensate for missing and/or noisy data and improve the accuracy of the outputted RoM measurements and scores.

To predict PsA flares, deep learning approaches will be used to fuse prospectively collected clinical (eg, age, sex, BMI, stress, fatigue, pain, medical history, treatment), biological (laboratory measurements), environmental (weather and air pollution) and digital data, with latter being monitored through smartphone and smartwatch passively (eg, typing sessions, accelerometer, physical activity, screen time, sleep patterns, heart rate) and through active video and photo tests.^49 50^ The deep learning paradigm of few-shot learning on foundation models trained on distinctly large datasets enables the adaptation of complex, deep models on the study population. Various levels of modality fusion ranging from early fusion of unprocessed data captured by the smartwatch and smartphone, to late fusion of derived (video, image, physical activity) features and digital biomarkers are explored to optimise the model’s sensitivity. The physician-reported flare will serve as the gold standard for flare identification; however, self-registered flare in the app, flare as detected by the digital biomarker and the patient-reported flare from questionnaire will also be explored. The goal is to identify predictive patterns that allow for early, individualised flare prediction. Model performance will be evaluated using AUC with various precision-recall cutoffs. For model development, data will be randomly split into training, validation and test sets, with flexibility to adjust based on dataset characteristics. Internal validation will be conducted using bootstrap and cross-validation techniques.

## Discussion

Patients with PsA often experience unpredictable changes in disease activity. However, routine clinical practice allows patients to discuss their symptoms with physicians only a few times per year during scheduled visits. Relying solely on routine visits limits our understanding of the disease and can lead to missed opportunities to adjust treatment, prevent flares or avoid long-term joint damage. Increasing the frequency of clinic visits or introducing additional online assessments to address this gap would impose a burden on both patients and physicians.

In this study, we aim to develop digital biomarkers for continuous, real-time and unobtrusive monitoring of PsA disease activity. There is currently no standard definition of a PsA flare.^32^ Previous studies on PsA flares mostly relied on patient- or physician-reported flare either based on recall of past events or assessed during predetermined follow-up visits, or disease activity scores.^32^ Our study adopts a comprehensive approach by prospectively collecting information on physician- and patient-reported flares during study visits and patient-initiated visits due to flares, real-time patient-reported flare registered in the app and disease activity scores. Moreover, the study includes a diverse PsA patient population from multiple centres and from four countries in real-world clinical settings.

In clinical practice, patients with PsA, even those with low disease activity, are at risk of flares. Besides, patients have an unmet need to better understand their disease, including factors that may trigger their flares. However, there is a gap in literature on the triggers of these flares.^32^ To the best of our knowledge, this is the first cohort designed to identify triggers of PsA flare among adult patients with PsA patients using multimodal data. These data include clinical, biological, environmental and digital measures collected prospectively.

This study may be limited by participant dropout, loss to follow-up and missingness in high-frequency data from smartphone and smartwatch. To mitigate these limitations, appropriate missing data handling and imputation methods will be applied. In addition, app updates, ongoing data quality checks and communication with participants and study sites will be used to identify and troubleshoot issues that may affect data collection. Second, self-reported questionnaires may be influenced by recall bias and socially desirable answers. However, objective measures will complement self-reported data (eg, hair cortisol levels as an indicator of stress, physician assessments of disease activity). Third, inclusion is limited to patients with smartphones, which may limit the generalisability of the findings among patients that do not have smartphones. Nevertheless, smartphone ownership is widespread, and the inclusion of participants from multiple sites across four countries increases the diversity and representativeness of the cohort. Finally, models developed in this study will be internally validated, thus external validation in independent cohorts will be required.

Currently, the integration of digital biomarkers into rheumatology care is limited. Findings from this study will provide valuable insights into the use of digital biomarkers for monitoring PsA disease activity, and the triggers of PsA flares. This knowledge will support the design of future interventions aimed at flare prevention and management, and empower patient-informed decision-making, resulting in better disease control.

### Ethics and dissemination

Ethical approval has been granted from the Medical Research Ethics Committee (MREC) of each of the participating countries (MREC Erasmus MC, the Netherlands (MEC-2023-0470); HRA and Health and Care Research Wales, UK (332916); NOVA Medical School|Faculdade de Ciências Médicas (NMS|FCM) Ethical Committee (CEFCM), Portugal (124/2023/CEFCM); MREC Hipokrateion Hospital Thessaloniki, Greece (5549/31.01.24)). For each of the local participating sites, institutional (and ethical) approval has also been granted.

We plan to disseminate the findings of this study through reports to the European Union funding body, updates on the iPROLEPSIS project website,^44^ newsletters provided to study participants and participating sites, outreach to the public community via social media, presentations at national and international conferences and symposiums and scientific publications.
