# Supplementary material for: Psoriatic arthritis digital phenotyping and inflammation drivers (PDPID) study: protocol for an international multicentre prospective cohort
Source: BMJ Open. 2026 Sep 28;16(9):e115903. doi: 10.1136/bmjopen-2025-115903 (PMC13629932; doi:10.1136/bmjopen-2025-115903)
Supplement: Supplementary file 1 [file bmjopen-2025-115903.reviewer_comments.pdf]

PEER REVIEW HISTORY

BMJ Open publishes all reviews undertaken for accepted manuscripts. Reviewers are asked to complete a checklist review form and are provided with free text boxes to elaborate on their assessment. These free text comments are reproduced below.

ARTICLE DETAILS

Title (Provisional)

Psoriatic arthritis digital phenotyping and inflammation drivers (PDPID) study: Protocol for an international multicenter prospective cohort

Authors

Hojeij, Batoul; Tchetverikov , I.; Vasileiou, Eleni; Apostolidis, George; Dimitroulas, Theodoros; Mytilinaiou, Maria; Gonçalves, Cátia; Rodrigues, Ana M; Coates, Laura; Konstantinidis, Dimitrios; Dimitropoulos, Kosmas; Melanitis, Nikos; Foolen, Jasper; Wagenaar, Wendy; Charisis, Vasileios; Hadjileontiadis, Leontios J; Luime, Jolanda J

VERSION 1 - REVIEW

**Reviewer**	**1**
**Name**	**Adebajo, Ade**
**Affiliation**	**Division of Genomic Medicine, Academic Department of Rheumatology**
**Date**	**28-Jan-2026**
**COI**	**None**

This protocol addresses an important research question of clinical relevance.

The Research Team is appropriate with relevant expertise.

It is unclear as to how the four countries were chosen.

The authors appropriately list the limitations. What measures are being out into place to mitigate against these limitations.

Do any of the patient research partners meet the criteria for authorship?

**Reviewer**	**2**
**Name**	**Gula, Zofia**
**Affiliation**	**Jagiellonian University**
**Date**	**05-Jul-2026**

COI	None

Overall, I find the study protocol well designed and have no major concerns. One point that may strengthen the study would be to clarify whether information on potential flare triggers, particularly recent infections, vaccinations and mechanical stress/trauma, will be collected prospectively. In addition, it would be useful to specify whether changes in antirheumatic treatment and concomitant medications during follow-up will be systematically recorded, as these factors may substantially influence flare occurrence and the interpretation of the collected biomarkers.

VERSION 1 - AUTHOR RESPONSE

Reviewer: 1 Dr. Ade Adebajo, Division of Genomic Medicine
Comments to the Author:
This protocol addresses an important research question of clinical relevance. The Research Team is appropriate with relevant expertise.
Thank you for reviewing the study protocol.

It is unclear as to how the four countries were chosen.
The four countries (the Netherlands, the United Kingdom, Portugal, and Greece) are participating as consortium partners in the iPROLEPSIS Horizon Europe project in which the study is embedded, representing North-Western and Southern Europe. This multicenter design increases the diversity of the study population including variation in patient characteristics and healthcare settings. We have also added this information to the manuscript.

Study design and population (p.8): “The study is conducted across 30 sites in four European countries (the Netherlands, UK, Portugal and Greece) participating in the iPROLEPSIS project[44] and representing North-Western and Southern Europe.”

The authors appropriately list the limitations. What measures are being out into place to mitigate against these limitations.
We have added the mitigation strategies against the limitations to the manuscript.

Discussion (p.24): “This study may be limited by participant dropout, loss to follow-up, and missingness in high frequency data from smartphone and smartwatch. To mitigate these limitations, appropriate missing data handling and imputation methods will be applied. In addition, app updates, ongoing data quality checks and communication with participants and study sites will be used to identify and troubleshoot issues that may affect data collection. Second, self-reported questionnaires may be influenced by recall

bias and socially desirable answers. However, objective measures will complement self-reported data (e.g., hair cortisol levels as an indicator of stress, physician assessments of disease activity). Third, inclusion is limited to patients with smartphones, which may limit the generalizability of the findings among patients that do not have smartphones. Nevertheless, smartphone ownership is widespread, and the inclusion of participants from multiple sites across four countries increases the diversity and representativeness of the cohort. Finally, models developed in this study will be internally validated, thus external validation in independent cohorts will be required."

Do any of the patient research partners meet the criteria for authorship?
Thank you for pointing this out. The development of the study idea builds on previous discussions with the patient research partners in the Netherlands. During the study design phase, the patient research partners also provided insights into the patient perspectives on certain aspects of the study (e.g., study assessments and app features). Considering their contribution, we have added two patient research partners as co-authors. They also reviewed and approved the final version of the manuscript.

Reviewer: 2 Dr. Zofia Gula, Jagiellonian University
Comments to the Author:
Overall, I find the study protocol well designed and have no major concerns.

Thank you for your positive evaluation of the study protocol.

One point that may strengthen the study would be to clarify whether information on potential flare triggers, particularly recent infections, vaccinations and mechanical stress/trauma, will be collected prospectively.
The study does not collect data on infections and vaccinations; however, several stress-related measures that may act as potential flare triggers are collected, including perceived stress questionnaire, major life events, and cortisol levels which are collected prospectively after patient inclusion in the study. Information on other flare triggers (e.g., BMI, pain, fatigue, sleep, stiffness) will be also collected prospectively. We have also clarified this information in the manuscript.

- Table 2 (p.14) provides an overview of all clinical variables collected in the study, including the timing of data collection for each variable.
- Objectives (p.7) "…(ii) machine learning models for flare prediction based on clinical, gut microbiome, genetic, and environmental data collected prospectively"

- Data analysis plan (p. 22) “ To predict PsA flares, deep learning approaches will be used to fuse prospectively collected clinical (e.g., age, sex, BMI, stress, fatigue, pain, medical history, treatment), biological (laboratory measurements), environmental (weather and air pollution) and digital data, with later being monitored through smartphone and smartwatch passively (e.g., typing sessions, accelerometer, physical activity, screen time, sleep patterns, heart rate) and through active video and photo tests.”
- Discussion (p.24): “These data include clinical, biological, environmental, and digital measures collected prospectively.”

In addition, it would be useful to specify whether changes in antirheumatic treatment and concomitant medications during follow-up will be systematically recorded, as these factors may substantially influence flare occurrence and the interpretation of the collected biomarkers.

Data on medication (anti rheumatic and non anti-rheumatic drugs) as well as dietary supplements is collected during each visit. We have clarified this information in manuscript.

- Questionnaires (p.13): “The questionnaires are administered at baseline and at each follow-up visit every 3 months covering general and clinical overview, medication (anti-rheumatic and non anti-rheumatic) and dietary supplements,...”
- Table 2 (p.14). Clinical collected measures of the PDPID study

**Collected measure**	**Parameter**	**Assessment period**					
		**T0**	**T3**	**T6**	**T9**	**T12**	**TFlare**
**Medication and supplement use**							
Anti-rheumatic drugs	Other	✓	✓	✓	✓	✓	
Non anti-rheumatic drugs	Other	✓	✓	✓	✓	✓	
Dietary supplements	Other	✓	✓	✓	✓	✓	
